# Upper Gastrointestinal Bleeding in Cirrhosis: Differential Performance of Risk Scores for In-Hospital Mortality and Intensive Care Unit Triage Decisions

**DOI:** 10.7759/cureus.104510

**Published:** 2026-03-01

**Authors:** Ilie Marius I Ciorba, Nicoleta Craciun Ciorba, Simona M Bataga

**Affiliations:** 1 Internal Medicine, George Emil Palade University of Medicine, Pharmacy, Science, and Technology of Târgu Mureș, Tîrgu Mureș, ROU; 2 Department of Medicine and Pharmacy, Institution Organizing University Doctoral Studies, University of Medicine, Pharmacy, Science and Technology “George Emil Palade”, Tîrgu Mureș, ROU; 3 Gastroenterology, George Emil Palade University of Medicine, Pharmacy, Science, and Technology of Târgu Mureș, Tîrgu Mureș, ROU

**Keywords:** aims65, cirrhosis, in-hospital mortality, intensive care unit, meld-na, risk stratification, triage, variceal bleeding

## Abstract

Background

Intensive care unit (ICU) case triage in cirrhotic-associated upper gastrointestinal bleeding (UGIB) varies greatly by local practice, while existing risk stratification data prioritizes mortality and rebleeding rather than ICU resource use.

Methods

We performed a retrospective cohort study of consecutive cirrhotic patients with UGIB in a tertiary referral center between January 1, 2024, and December 31, 2025. Our primary endpoint was in-hospital mortality, and a secondary endpoint was ICU triage decision. The institutional ICU criteria included persistent hypotension after initial fluid resuscitation (systolic blood pressure (SBP) <90 mmHg and/or vasopressor requirement), respiratory distress requiring advanced support (intubation or escalating oxygen requirement) or severe metabolic acidosis (pH ≤7.20 and/or lactate ≥4 mmol/L). We compared discrimination (Area under the receiver operating characteristic curve (AUROC), with bootstrap 95% confidence interval (CI)) of UGIB scores (Albumin, International Normalized Ratio, Mental status, Systolic blood pressure, age 65 (AIMS65); Rockall and Glasgow-Blatchford) and liver-specific scores (Model for End-stage Liver Disease with Sodium (MELD-Na), Child-Pugh, Albumin to Bilirubin index (ALBI), Platelet-Albumin-Bilirubin index (PALBI), International Normalized Ratio to Albumin (PTAR), Aspartate Aminotransferase (AST) to platelet ratio index (APRI)). Logistic regression models assessed incremental value of combined physiology and liver severity models. A prespecified sensitivity analysis restricted the cohort to endoscopically confirmed variceal bleeding.

Results

The cohort included 224 patients with a median age 58 years and 174 (77.7%) were men. Variceal bleeding was present in 212 (94.6%) cases. ICU admission occurred in 37 (16.5%) cases and in-hospital mortality occurred in 53 (23.7%) cases. For mortality, MELD-Na showed the highest discrimination (AUROC 0.898, 95% CI 0.849-0.940), followed by PTAR (0.865) and ALBI (0.852). For ICU admission, AIMS65 (0.930, 95% CI 0.896-0.960) and Rockall (0.919, 95% CI 0.885-0.949) outperformed liver-only scores. A combined MELD-Na + AIMS65 model improved mortality discrimination (AUROC 0.919) and calibration versus either score alone.

Conclusions

In cirrhosis-associated UGIB, liver dysfunction severity (estimated through MELD-Na and related scores) best predicts in-hospital mortality, while acute physiology (AIMS65 and Rockall scores) best predicts ICU admission. Combining physiology and liver severity improved mortality prediction. ICU analyses reflect prediction of observed triage decisions under institutional criteria and require external validation against organ-support endpoints.

## Introduction

Cirrhosis-associated upper gastrointestinal bleeding (UGIB) cases, commonly due to esophageal and gastric variceal hemorrhage, remains a major cause of preventable in-hospital death and resource utilization. Current guidelines emphasize early resuscitation, vasoactive therapy, antibiotics and urgent endoscopic hemostasis [1-5]. However, bedside decisions regarding Intensive Care Unit (ICU) admission are less standardized, and ICU admissions frequently reflect institutional practice patterns and capacity constraints, rather than standardized norms of practice [6].

Risk stratification research in variceal bleeding has been usually focused on short-term mortality and rebleeding, typically using Child-Pugh or Model for End-stage Liver Disease (MELD)-based severity evaluation and endoscopic findings [7]. On the other hand, general UGIB scores such as Albumin, International normalized ratio (INR), Mental Status, Systolic blood pressure, Age 65 (AIMS65), Rockall and Glasgow-Blatchford (GBS) incorporate acute physiology and have been evaluated for adverse outcomes and ICU decisions in mixed UGIB populations [8-11]. Whether these physiology-based UGIB scores better predict ICU admission in cirrhosis-associated bleeding and how they compare with liver-specific scores (including laboratory-derived scores such as Albumin to Bilirubin index (ALBI), Platelet-Albumin-Bilirubin index (PALBI), and International Normalized Ratio to Albumin ratio (PTAR)), remains insufficiently addressed.

## Materials and methods

We compared commonly used UGIB scores and liver-specific scores for two clinically distinct outcomes: in-hospital mortality and ICU admission, because accurately anticipating ICU need can support timely escalation for unstable patients while avoiding potentially unnecessary ICU admissions and optimizing constrained critical-care resources. We further tested whether combining liver dysfunction severity (MELD-Na) with acute physiology (AIMS65) improved predictive performance for mortality. Laboratory examinations were performed using Sysmex XN-1000 system (Sysmex Europe, Hamburg, Germany) for hematological determination, respectively Cobas Pure e402 system (Roche Diagnostics, Rotkreuz, Switzerland) for the other biochemical markers. Endoscopy was performed by using an Olympus Evis Exera II 185 HD standard gastroscope (Olympus Corporation, Tokyo, Japan), after getting patient written consent for endoscopy, standardized by hospital procedure.

Study design and setting

**Figure 1:**
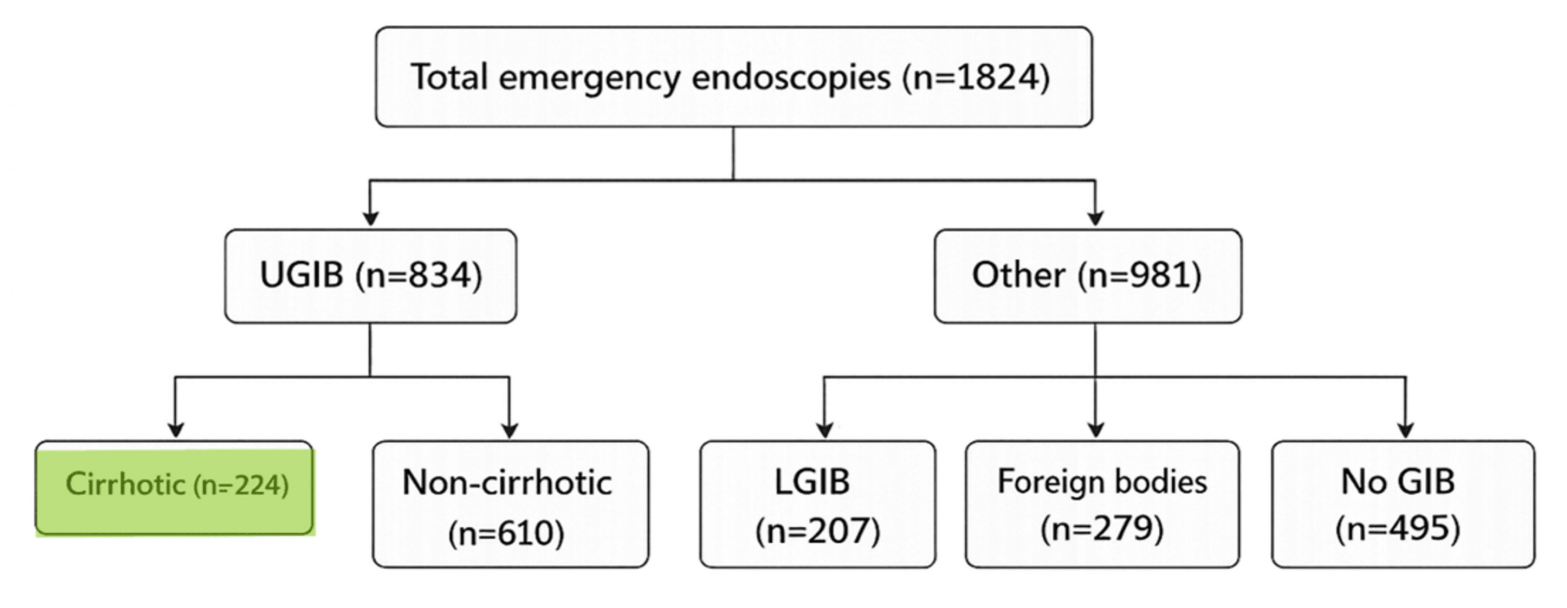
Cohort selection from the total number of emergency endoscopies performed UGIB: upper gastrointestinal bleed; LGIB: lower gastrointestinal bleed; GIB: gastrointestinal bleed.

This retrospective cohort study included consecutive adult patients admitted for cirrhosis-associated UGIB in a single tertiary referral center between January 1, 2024 and December 31, 2025. During the study period, a total of 1824 emergency endoscopies were performed, diagnosing 834 UGIB, 207 lower GI bleeds and 495 cases with no bleeding or performed for other diagnosis suspicion (refractory or severe emesis, gastroparesis or outlet obstruction, severe anemia), as presented in Figure 1.

Data were obtained from electronic patient charts (including vital signs, laboratory results, and transfusion records) and corroborated against endoscopy reports. Urgent upper endoscopy was performed in all patients with a median time-to-endoscopy of 4.9 hours (Interquartile range or IQR 2.8-5.4) and all procedures occurring within six hours (n=224/224, 100%, hospital protocol, 24/7 on call endoscopy team). Time-to-endoscopy was defined as the interval from emergency department admission timestamp to procedure start time (endoscope insertion) recorded in the endoscopy report.

Participants

Inclusion criteria were set to patients of 18 years or older, liver cirrhosis previously diagnosed or newly established at presentation (abdominal ultrasound consistent with cirrhosis and bloodwork consistent with chronic liver disease) and acute UGIB with upper endoscopy performed during the admission. We did not exclude non-variceal causes of UGIB in cirrhotic patients and variceal bleeding status was derived from endoscopic conclusions. We queried all admissions coded for UGIB and endoscopy during the study period and included those meeting cirrhosis criteria. The unit of analysis was the patient, repeat presentations being identified using the personal identification number as recorded in the hospital information system. For patients with multiple eligible presentations, only the first episode was retained (screened n=224; included n=224; analyzed n=224, 100%). For patients transferred from outside hospitals, baseline lab values were defined as the first bloodwork results available from the referring unit. Pre-hospital fluid resuscitation was recorded. No pre-hospital blood transfusions were administered and initial physiologic measurements were interpreted in the context of the documented resuscitation volume administered before arrival.

Endpoints

The primary endpoint was set as in-hospital mortality. The secondary endpoint was set as ICU admission during the index hospitalization (the observed ICU triage decisions under institutional criteria).

ICU admission criteria

According to the institutional practice during the study period, ICU admission was indicated for any of the following criteria at presentation or during early hospitalization: severe shock defined as unresponsive hypotension after initial fluid resuscitation (systolic blood pressure (SBP) <90 mmHg) and/or need for vasopressor support, respiratory distress requiring advanced support or airway protection (endotracheal intubation, non-invasive ventilation or oxygen requirement ≥6 L/min by nasal cannula or FiO2 ≥0.40 to maintain SpO2 ≥92%) or severe metabolic acidosis defined as pH ≤7.20 and/or lactate ≥4 mmol/L. [4-6,12,13]. Final triage decisions were made by the on-call intensivist and gastroenterology team. Post-hoc, ICU admissions were adjudicated by two clinicians (one intensivist and one gastroenterologist) through chart review to confirm one criterion or more was met at emergency department presentation or within the first 24 hours of hospitalization, while disagreements were resolved by consensus. For clarity, ICU admission was analyzed as an observed triage decision under these criteria and does not necessarily represent ICU-level organ support requirements.

Risk scores

We evaluated liver-specific scores (MELD-Na, Child-Pugh class, ALBI, PALBI, PTAR, aspartate aminotransferase-to-platelet ratio Index (APRI), bilirubin to albumin ratio (BAR) and UGIB scores (AIMS65, Rockall, Glasgow-Blatchford) as recorded in the database, using the original published definitions. Rockall was calculated and used as the full Rockall score (including endoscopic diagnosis and stigmata) [8-10,12-14].

Statistical analysis

Continuous variables are presented as median (interquartile range, IQR) and categorical variables as n (%). Group comparisons used Mann-Whitney U tests for continuous variables and Fisher exact or chi-square tests for categorical variables. Endpoints and all prespecified risk scores used for primary analyses were complete. All component variables required to compute prespecified scores were available for all included admissions. Therefore, no imputation was performed. Discrimination was assessed with area under the receiver operating characteristic curve (AUROC) and 95% CIs derived by bootstrap resampling (2000 iterations). We built logistic regression models for mortality and ICU admission and reported odds ratios (OR) with 95% confidence intervals. Calibration was assessed using calibration plots and by reporting the calibration intercept and slope. Internal validation used bootstrap optimism correction (1000 resamples) for AUROC, Brier score, and calibration parameters. We performed decision-curve analysis to quantify net benefit across clinically plausible threshold probabilities. Reporting followed strengthening the reporting of observational studies (STROBE) guidance [15] for observational studies and prediction reporting was aligned with transparent reporting of a multivariable individual prognosis or diagnosis model (TRIPOD) [16] principles.

Data collection, storage and preliminary analysis of the database was performed using Microsoft Excel (Microsoft Corp., Redmond, WA, USA). Statistical analyses were performed using Python (Python Software Foundation, Python Language Reference, v3.11.7, pandas v2.2.2, NumPy v1.26.4, SciPy v1.11.4, statsmodels v0.14.2, scikit-learn v1.4.2, matplotlib v3.8.4) and R (version 4.5.2, R Foundation for Statistical Computing, Vienna, Austria).

Ethics

This study was conducted in accordance with the Declaration of Helsinki and institutional standards for research involving human participants. The protocol was reviewed and approved by the Mureș County Emergency Clinical Hospital Medical Ethics Commission for Clinical studies (approval number Ad 34800). Because this was a retrospective analysis of routinely collected clinical data with minimal risk to participants, the requirement for informed consent was waived as determined by the reviewing board. All data were de-identified prior to analysis and handled in accordance with applicable data protection policies.

## Results

Cohort characteristics

The cohort included a total of 224 patients. Median age was 58 years (51-68) and 174 (77.7%) were men. Alcohol-related cirrhosis accounted for 173 (77.2%) of all cases. Variceal bleeding was present in 94.6% (212) of cases. ICU admission occurred in 16.5% (37) of cases and in-hospital mortality was recorded at 23.7% (53). Hospital course characteristics stratified by survival status are shown in Table 1.

**Table 1:** Hospital course characteristics and management, overall and stratified by in-hospital mortality Values are median (interquartile range) or n (%); MASLD: metabolic dysfunction-associated steatotic liver disease; MELD-Na: Model for End-Stage Liver Disease - Sodium; AIMS65: Albumin, INR, Mental Status, Systolic Blood pressure, 65 (age); ALBI: Albumin to bilirubin index; PALBI: Platelet-Albumin-Bilirubin index; PTAR: INR-to-albumin ratio; INR: international normalized ratio; ICU: intensive care unit.

Characteristic	Overall (n=224)	Survivors (n=171)	Non-survivors (n=53)
Age, years	58.0 (51.0-68.0)	59.0 (52.0-68.0)	57.0 (49.0-69.0)
Male sex	174 (77.7%)	132 (77.2%)	42 (79.2%)
Etiology: Alcohol-related	173 (77.2%)	127 (74.3%)	46 (86.8%)
Etiology: Alcohol + viral	12 (5.4%)	10 (5.8%)	2 (3.8%)
Etiology: Viral	13 (5.8%)	11 (6.4%)	2 (3.8%)
Etiology: MASLD	14 (6.2%)	11 (6.4%)	3 (5.7%)
Etiology: Autoimmune	9 (4.0%)	9 (5.3%)	0 (0.0%)
Etiology: Vascular/other	3 (1.3%)	3 (1.8%)	0 (0.0%)
Variceal bleeding (endoscopic)	212 (94.6%)	163 (95.3%)	49 (92.5%)
MELD-Na	18.0 (13.0-24.0)	16.0 (12.0-21.0)	31.0 (23.0-35.0)
Child-Pugh class A	30 (13.4%)	30 (17.5%)	0 (0.0%)
Child-Pugh class B	100 (44.6%)	93 (54.4%)	7 (13.2%)
Child-Pugh class C	94 (42.0%)	48 (28.1%)	46 (86.8%)
AIMS65	2.0 (1.0-3.0)	2.0 (1.0-3.0)	4.0 (3.0-4.0)
ALBI score	-1.5 (-2.0 - -0.9)	-1.7 (-2.1 - -1.2)	-0.9 (-1.2 - -0.5)
PALBI score	-1.9 (-2.2 - -1.4)	-2.0 (-2.4 - -1.6)	-1.4 [-1.7 - -1.3)
PTAR	0.5 (0.4-0.7)	0.4 [0.4-0.6]	0.8 (0.7-1.3)
Hemoglobin, g/dL	6.8 (6.0-7.9)	6.8 (6.2-8.0)	6.6 (5.7-7.3)
INR	1.4 (1.2-1.8)	1.4 (1.2-1.6)	2.0 (1.7-3.1)
Creatinine, mg/dL	0.8 (0.7-1.4)	0.8 [0.6-1.0]	1.8 (1.1-2.9)
Albumin, g/dL	2.9 (2.5-3.3)	3.1 (2.7-3.4)	2.5 (2.1-2.8)
Total bilirubin, mg/dL	2.0 (0.8-3.7)	1.6 (0.7-2.8)	5.1 (2.0-8.2)
Hepatocellular carcinoma	21 (9.4%)	12 (7.0%)	9 (17.0%)
Portal vein thrombosis	30 (13.4%)	19 (11.1%)	11 (20.8%)
Antiplatelet therapy	23 (10.3%)	20 (11.7%)	3 (5.7%)
Terlipressin administered	193 (86.2%)	148 (86.5%)	45 (84.9%)
ICU admission	37 (16.5%)	15 (8.8%)	22 (41.5%)
Endoscopic therapy: Band ligation	151 (67.4%)	117 (68.4%)	34 (64.2%)
Endoscopic therapy: Argon plasma coagulation	25 (11.2%)	19 (11.1%)	6 (11.3%)
Endoscopic therapy: No endoscopic therapy	37 (16.5%)	29 (17.0%)	8 (15.1%)
Endoscopic therapy: Balloon tamponade	2 (0.9%)	0 (0.0%)	2 (3.8%)
Endoscopic therapy: Injection + clip	3 (1.3%)	3 (1.8%)	0 (0.0%)
Endoscopic therapy: Clip	3 (1.3%)	3 (1.8%)	0 (0.0%)
Endoscopic therapy: Injection	2 (0.9%)	0 (0.0%)	2 (3.8%)
Endoscopic therapy: Band ligation + balloon	1 (0.4%)	0 (0.0%)	1 (1.9%)

Discrimination of risk scores for mortality and ICU admission

**Figure 2:**
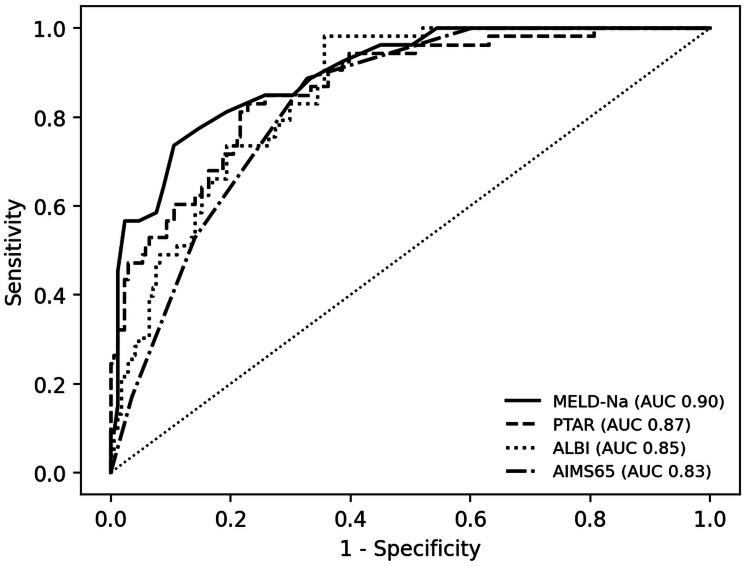
Receiver operating characteristic curves for in-hospital mortality using selected scores AUC: area under the curve; MELD-Na: Model for End-Stage Liver Disease - Sodium; PTAR: International Normalized Ratio-to-Albumin ratio; ALBI: Albumin to Bilirubin index; AIMS65: Albumin, INR (international normalized ratio), Mental Status, Systolic blood pressure, 65 (age).

Across evaluated scores, mortality was most strongly associated with liver dysfunction severity. MELD-Na presented the highest discrimination for in-hospital mortality (AUROC 0.898, 95% confidence interval, CI, 0.849-0.940), followed by PTAR and ALBI scores (Table 2, Figure 2).

**Table 2:** Discrimination (AUROC) of risk scores for in-hospital mortality and ICU admission with bootstrap 95% confidence intervals. Child-Pugh was analyzed as ordinal class (A=1, B=2, C=3) for discrimination analyses; AUROC: area under the receiver operating characteristic curve; MELD-Na: Model for End-Stage Liver Disease - Sodium; AIMS65: Albumin, INR, Mental Status, Systolic blood pressure, 65 (age); ALBI: Albumin to bilirubin index; PALBI: Platelet-Albumin-Bilirubin index; PTAR: International Normalized Ratio-to-albumin ratio; GBS: Glasgow-Blatchford score; APRI: Aspartate aminotransferase to Platelet Ratio Index; BAR: bilirubin to albumin ratio.

Score	AUROC for in-hospital mortality (95% CI)	AUROC for ICU admission (95% CI)
MELD-Na score	0.898 (0.850-0.941)	0.791 (0.715-0.856)
Child-Pugh class	0.805 (0.751-0.854)	0.848 (0.817-0.881)
ALBI score	0.852 (0.800-0.900)	0.763 (0.688-0.830)
PALBI score	0.806 (0.741-0.865)	0.733 (0.656-0.804)
PTAR	0.865 (0.806-0.916)	0.794 (0.720-0.857)
AIMS65	0.829 (0.773-0.876)	0.930 (0.895-0.959)
Rockall score	0.615 (0.525-0.705)	0.919 (0.883-0.948)
GBS	0.703 (0.623-0.773)	0.763 (0.687-0.827)
APRI	0.755 (0.680-0.827)	0.669 (0.567-0.757)
BAR	0.788 (0.713-0.853)	0.728 (0.631-0.814)

**Figure 3:**
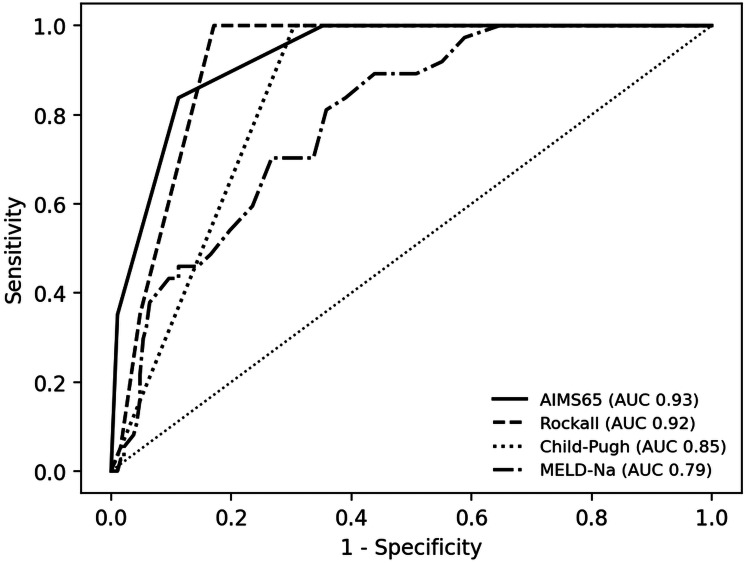
Receiver operating characteristic curves for ICU admission using selected scores. AUC: area under the curve; MELD-Na: Model for End-stage Liver Disease - Sodium.

In contrast to these results, ICU admission was best predicted by the AIMS65 score (AUROC 0.930, 95% CI 0.896-0.960) while full Rockall score (AUROC 0.919, 95% CI 0.885-0.949) outperformed liver-only scores for this endpoint (Table 2, Figure 3).

Multivariable models and incremental value of combining physiology with liver severity

**Figure 4:**
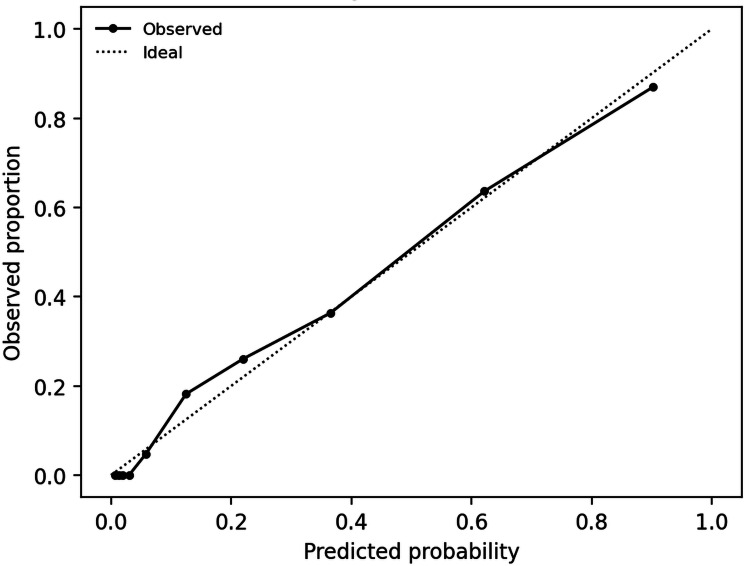
Calibration of the combined MELD-Na + AIMS65 mortality model MELD-Na: Model for End-stage Liver Disease - Sodium; AIMS65: Albumin, INR (international normalized ratio), Mental Status, Systolic blood pressure, 65 (Age).

When applying univariable logistic regression, both MELD-Na and AIMS65 were associated with in-hospital mortality. A combined MELD-Na with AIMS65 model improved mortality discrimination (AUROC 0.919, optimism-corrected AUROC 0.917) and reduced Brier score (optimism-corrected 0.101) compared with either score alone (Table 3, Figure 4).

**Table 3:** Logistic regression models for in-hospital mortality and ICU admission Odds Ratios (ORs) are per 1-point increase in score; AUROC: Area under the receiver operating characteristic curve; CI: confidence interval; MELD-Na: Model for End-stage Liver Disease - Sodium; AIMS65: Albumin, INR (International normalized ratio), Mental Status, Systolic blood pressure, 65 (age); ICU: Intensive Care Unit.

Outcome	Model	Predictor	OR (95% CI)	p value	AUROC	Brier
In-hospital mortality	M1: MELD-Na	MELD-Na score	1.26 (1.18-1.34)	<0.001	0.898	0.101
In-hospital mortality	M2: AIMS65	AIMS65 score	2.83 (2.04-3.92)	<0.001	0.829	0.138
In-hospital mortality	M3: MELD-Na + AIMS65	MELD-Na score	1.21 (1.13-1.30)	<0.001	0.919	0.097
In-hospital mortality	M3: MELD-Na + AIMS65	AIMS65 score	1.86 (1.26-2.74)	0.002	0.919	0.097
ICU admission	M4: AIMS65	AIMS65 score	8.77 (4.32-17.81)	<0.001	0.930	0.073
ICU admission	M5: Rockall	Rockall score	4.76 (2.78-8.15)	<0.001	0.919	0.090
ICU admission	M6: AIMS65 + Rockall	AIMS65 score	5.78 (2.80-11.90)	<0.001	0.963	0.058
ICU admission	M6: AIMS65 + Rockall	Rockall score	3.61 (1.83-7.15)	<0.001	0.963	0.058

**Figure 5:**
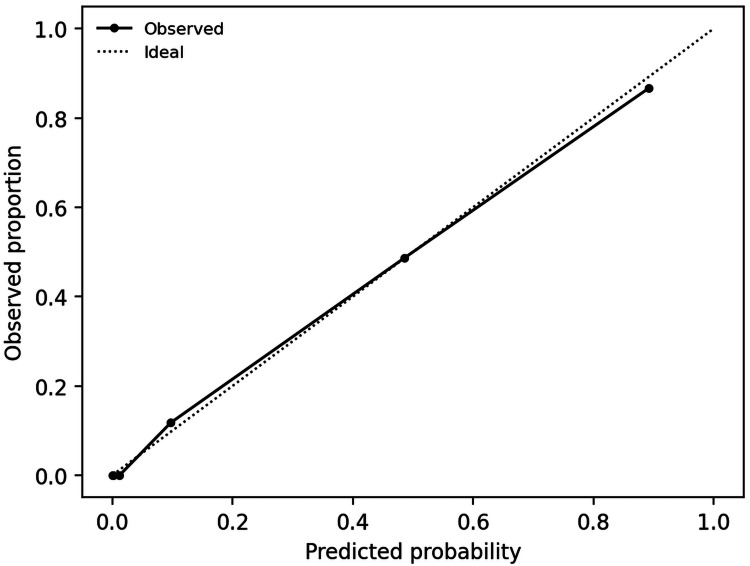
Calibration of the AIMS65 model for ICU admission AIMS65: Albumin, INR (international normalized ratio), Mental Status, Systolic blood pressure, 65 (age); ICU: Intensive Care Unit.

After optimism correction, calibration of the combined mortality model remained close to ideal (intercept -0.016, slope 0.958). For ICU admission, AIMS65 and full Rockall score were each strongly associated with ICU admission and the combined AIMS65 with Rockall model further improved discrimination (AUROC 0.963, optimism-corrected AUROC 0.962) and Brier score (optimism-corrected 0.061) (Table 3, Figure 5).

For ICU triage decisions, a combined AIMS65 + Rockall model provided the best discrimination (AUROC 0.963, Brier score 0.058).

For each logistic regression model, the predicted risk was calculated as p = 1 / (1 + exp(-(α + β1×X1 + β2×X2))). Table 4 reports regression coefficients (β, log-odds per one-point increase in score) and model intercepts (α) to enable external validation and bedside risk calculation.

**Table 4:** Logistic regression coefficients (log-odds) and intercepts for risk calculation. MELD-Na: Model for End-stage Liver Disease - Sodium; AIMS65: Albumin, INR (international normalized ratio), Mental Status, Systolic blood pressure, 65 (age); ICU: Intensive Care Unit.

Model	Predictors	Coefficients (β)	Intercept (α)
M1: MELD-Na (mortality)	MELD-Na	MELD-Na: 0.232	-6.314
M2: AIMS65 (mortality)	AIMS65	AIMS65: 1.039	-4.073
M3: MELD-Na + AIMS65 (mortality)	MELD-Na, AIMS65	MELD-Na: 0.192; AIMS65: 0.619	-7.239
M4: AIMS65 (ICU triage)	AIMS65	AIMS65: 2.171	-8.742
M5: Rockall (ICU triage)	Rockall	Rockall: 1.560	-13.193
M6: AIMS65 + Rockall (ICU triage)	AIMS65, Rockall	AIMS65: 1.754; Rockall: 1.285	-17.184

Decision-curve analysis

**Figure 6:**
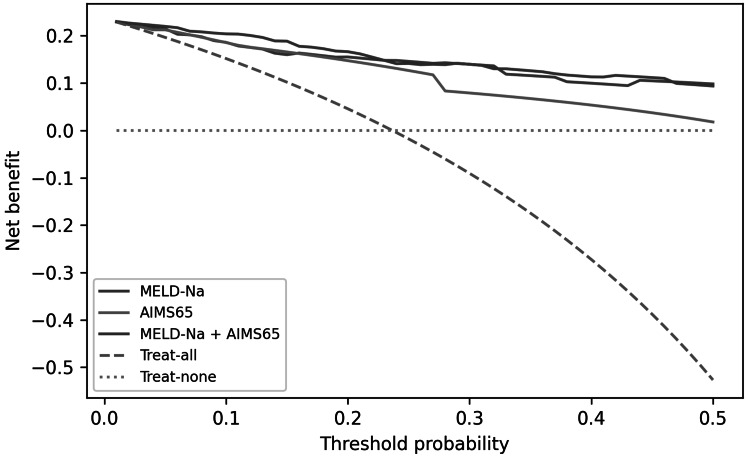
Decision-curve analysis for in-hospital mortality comparing MELD-Na, AIMS65, and the combined MELD-Na+AIMS65 model MELD-Na: Model for End-stage Liver Disease - Sodium; AIMS65: Albumin, INR (international normalized ratio), Mental Status, Systolic blood pressure, 65 (age).

**Figure 7:**
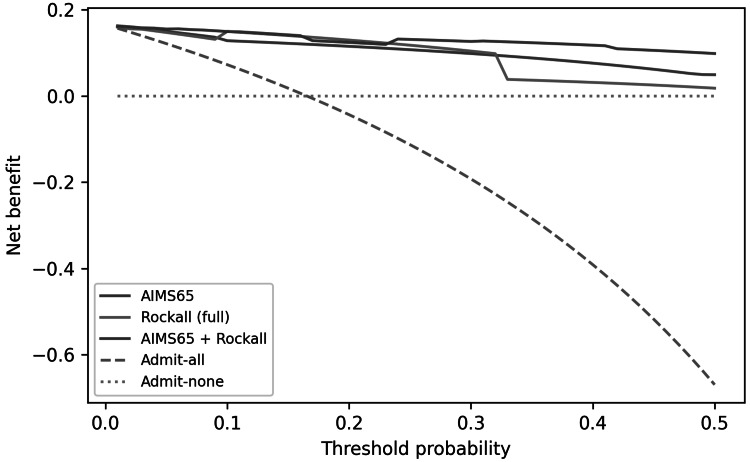
Decision-curve analysis for ICU admission comparing AIMS65, full Rockall, and the combined AIMS65 + Rockall model ICU: Intensive Care Unit; AIMS65: Albumin, INR (international normalized ratio), Mental Status, Systolic blood pressure, 65 (age).

Decision-curve analysis (DCA) suggested that physiology-based models provide higher net benefit across a range of clinically plausible threshold probabilities for predicting ICU triage decisions, while liver severity models provide higher net benefit for in-hospital mortality (Figures 6, 7).

Using AIMS65 at admission, thresholds in the 10-20% predicted ICU-risk range provided favorable net benefit compared with “admit-all” or “admit-none” strategies. These findings should be interpreted as decision-analytic evidence for predicting institutional triage decisions rather than as estimates of ICU level organ support, because ICU admission followed protocolized physiologic thresholds and because we could not measure hard ICU interventions (mechanical ventilation, vasopressors, renal replacement therapy) in this dataset. Therefore, any implementation language should be viewed as hypothesis generating and prospective validation should benchmark these models against organ-support endpoints and standard six-week outcomes.

Sensitivity analysis for the variceal bleeding subset

In the endoscopically confirmed variceal bleeding subset (n=212), results were consistent. MELD-Na remained the best discriminator for mortality (AUROC 0.900) while AIMS65 and Rockall remained the best discriminators for ICU admission (AUROC 0.928 and 0.917, respectively). The sensitivity analysis is described in the Appendix.

## Discussion

In this single-center cohort of cirrhosis-associated UGIB patients, managed in a tertiary referral center, we found that the severity of liver dysfunction and acute physiological distress both carried strong prognostic information but for different endpoints. MELD-Na achieved the highest discrimination for in-hospital mortality (AUROC ~0.90), while AIMS65 achieved the highest discrimination for ICU admission (AUROC ~0.93). These findings come in support of a two-axis view of risk in cirrhotic bleeding (chronic hepatic reserve and acute systemic instability) and suggest that endpoint choice (mortality vs. ICU admission) materially influences which score performs best.

Our mortality results align with prior variceal bleeding studies that emphasize liver disease severity as a dominant determinant of short-term outcomes. Reverter et al. recalibrated MELD to predict six‑week mortality after acute variceal bleeding and demonstrated that higher MELD values identify clinically meaningful risk strata (MELD ≥19 associated with ≥20% six‑week mortality) [17]. Similarly, Baveno VII and European Society for Gastrointestinal Endoscopy (ESGE) guidance recommend risk stratification using Child-Pugh and MELD in acute variceal hemorrhage, reflecting the consistent association between hepatic reserve and early mortality [1,2]. Our high MELD‑Na discrimination for in-hospital mortality is therefore biologically, and guideline-concordant. Moreover, the observed thresholds are within the range reported in prior series [1-3,17].

In contrast, ICU admission is a composite of physiology, anticipated clinical evolution and institutional practice. Importantly, because ICU admission in our center follows protocolized physiologic thresholds, performance for the ICU endpoint should be interpreted primarily as prediction of triage-rule concordance rather than purely independent prognostication. In mixed UGIB cohorts, AIMS65 has shown good performance for adverse outcomes and has been reported to outperform GBS and pre-endoscopy Rockall for predicting the need for ICU care [8,12]. Robertson et al. (n=424) found AIMS65 superior to GBS and pre-Rockall for predicting inpatient mortality and ICU admission, while Thandassery et al. also supported AIMS65 as a pragmatic early risk tool [12,18]. Our data extend these observations to a cirrhosis focused population, where physiology-based scores appear to better reveal the drivers of ICU triage (shock, altered mentation and cardiorespiratory distress), whereas liver-only scores (such as MELD-Na) remain more linked to mortality risk.

Notably, the full Rockall score showed limited discrimination for in-hospital mortality in our cohort (AUROC 0.615). This likely reflects differences between the score’s original population and a cirrhosis focused, predominantly variceal cohort. Full Rockall incorporates endoscopic diagnosis and stigmata of recent hemorrhage, which add prognostic value in mixed UGIB populations [9]. However, in cirrhotic patients with UGIB, early mortality is often determined more by hepatic reserve, decompensation (infection, acute-on-chronic liver failure) and complications of portal hypertension than by endoscopic lesions alone [19]. Moreover, near-uniform urgent endoscopy and a high prevalence of portal-hypertensive related bleeding can compress variability in Rockall’s diagnostic and stigmata components, attenuating its ability to separate mortality risk compared with MELD-based measurements [20].

Of the liver-derived laboratory scores, ALBI and PTAR performed strongly for mortality in our cohort. ALBI was originally developed as an objective liver function measure and has subsequently been applied beyond hepatocellular carcinoma settings [13]. In a large cirrhotic variceal bleeding cohort, Elshaarawy et al. reported good prognostic performance for ALBI and PALBI in predicting early mortality and rebleeding [14]. Coagulation-to-albumin ratios have also been proposed as rapid markers of bleeding severity and overall physiologic stress. In a large critical-care gastrointestinal bleeding cohort, Yang et al. reported that the PTAR was associated with mortality and demonstrated prognostic discrimination [21]. Together, these reports and our findings suggest that simple laboratory scores showing dysfunction and coagulopathy may approximate the prognostic signal of MELD-Na in the inpatient setting while remaining easy to compute.

We also observed that combining MELD-Na with AIMS65 incrementally improved discrimination for in-hospital mortality compared with MELD‑Na alone, consistent with the concept that “acute-on-chronic” stress adds additional mortality risk beyond baseline hepatic reserve. This combined model may help quantify risk in future external validation studies, but any operational use should remain cautious because it was derived and internally validated in a single-center cohort and because post-discharge outcomes were not captured.

ICU admission criteria for UGIB are not universally standardized but most guidelines converge on hemodynamic instability, need for ventilatory support and evolving organ failure as common triggers for higher-acuity care [4-6]. In variceal bleeding patients, guidelines emphasize resuscitation and airway protection in severe bleeding or impaired mental status with recent reviews explicitly noting ICU-level admission for suspected acute variceal bleeding in many settings [3,22,23]. Our prespecified institutional criteria (shock, respiratory distress, severe acidosis) map closely onto these higher-acuity triggers and help interpret why physiology-based scores that include hypotension, mental status and biochemical disorder track ICU admission so well [6,7,11].

Limitations

Firstly, this is a single-center retrospective study conducted in a tertiary referral center. Population characteristics, acuity, and ICU thresholds may differ across institutions and health systems. We view this as a strength for real-world decision support in a tertiary setting but generalizability requires external validation. Secondly, our ICU admission definition shares constructs with acute physiology scores. Therefore, ICU discrimination estimates should be interpreted primarily as prediction of triage-rule concordance under institutional criteria rather than as independent prognostication. Even with prespecified physiologic thresholds and post-hoc adjudication, ICU admission may also reflect bed availability and local capacity constraints. Thirdly, the database was limited to in-hospital outcomes and did not capture standard six-week endpoints (mortality or rebleeding). In our regional network, many patients complete six-week follow-up in their local centers rather than returning to the tertiary center, making in-hospital mortality a pragmatic operational endpoint for triage and inpatient quality improvement. To reduce overfitting, given the number of ICU and mortality events, combined models were intentionally parsimonious (two predictors) and internally validated using bootstrap optimism correction. Finally, although we used bootstrap internal validation with optimism correction, independent external validation is required before any clinical deployment.

## Conclusions

In cirrhosis-associated UGIB, liver dysfunction severity (captured best by MELD-Na and related liver-specific scores) showed the strongest discrimination for in-hospital mortality, whereas acute physiology scores (AIMS65 and the full Rockall score) best predicted ICU triage decisions under our institutional criteria. These findings support a two-axis framing of risk in cirrhotic bleeding, where chronic hepatic reserve primarily drives mortality risk while acute systemic instability drives escalation of care. Combining MELD-Na with AIMS65 improved mortality prediction beyond either score alone, suggesting incremental value in integrating baseline liver severity with acute physiologic derangement.

ICU admission in this study reflects observed protocolized triage decisions and local capacity-dependent practice rather than a direct measure of the ICU level organ support, and we could not benchmark predictions against hard ICU interventions (non-invasive or mechanical ventilation, vasopressors, renal replacement therapy) or standard six-week outcomes. Accordingly, ICU-related discrimination should be interpreted as a prediction of triage-rule concordance. Prospective multicenter validation is needed to confirm transportability across different ICU thresholds and to evaluate whether combined liver-severity and physiology models improve decision-making when anchored to organ-support endpoints and longer-term outcomes before any clinical deployment.

## Appendices

Statistical appendix

Dataset and Endpoints

Analyses were performed on the study database (n=224, 100% admissions) extracted from the institutional electronic records. The primary cohort included all adult patients with cirrhosis admitted with upper gastrointestinal bleeding (UGIB), regardless of endoscopic source.

The primary endpoint was in-hospital mortality (death during index hospitalization) with operationalization in the database: In-Hospital Survival=0.

The secondary endpoint was ICU admission during the index hospitalization, interpreted as the observed ICU triage decision under institutional criteria with operationalization in the database: ICU=1.

The observed event counts were mortality 53/224 (23.7%), ICU triage decision 37/224 (16.5%).

Sensitivity Analysis Cohort (Variceal Bleeding Subset)

A prespecified sensitivity analysis restricted the cohort to admissions with endoscopically confirmed variceal bleeding. Variceal bleeding was defined by endoscopy diagnosis fields containing esophageal or gastric varices. The variceal subset size was 212/224 (94.6%).

Predictors and Score Inputs

Risk scores were taken directly from the database as recorded at initial evaluation (continuous totals): MELD-Na (Model for End-stage Liver Disease - Sodium), AIMS65 (Albumin, INR - International standardized ratio, Mental Status, Systolic blood pressure, age 65), and full Rockall score. No imputation was required for these variables (0 missing values for each).

Model Definitions

Six prespecified logistic regression models were fit at the admission level. Let p denote the predicted probability of the endpoint for a given admission. For each model: p = 1 / (1 + exp(−η)), where η = α + Σ βk·Xk.

Here α is the intercept and βk are log-odds coefficients for predictors Xk (Table 5).

**Table 5:** Model definitions MELD-Na: Model for End-stage Liver disease - Sodium; AIMS65: Albumin, INR (international normalized ratio), Mental Status, Systolic blood pressure, 65 (age); ICU: Intensive Care Unit.

Model	Outcome	Predictors
M1	In-hospital mortality	MELD-Na
M2	In-hospital mortality	AIMS65
M3	In-hospital mortality	MELD-Na + AIMS65
M4	ICU triage decision	AIMS65
M5	ICU triage decision	Rockall
M6	ICU triage decision	AIMS65 + Rockall

Coefficient Estimates and Risk Calculation

Table 6 reports fitted coefficients from maximum-likelihood logistic regression on the full cohort.

**Table 6:** Coefficient estimates and risk calculation MELD-Na: Model for End-stage Liver disease - Sodium; AIMS65: Albumin, INR (international normalized ratio), Mental Status, Systolic blood pressure, 65 (age); ICU: Intensive Care Unit; SE: standard error; CI: confidence interval; OR: Odds Ratio.

Model	Term	α/β	SE	OR	95% CI (OR)
M1	Intercept (α)	-6.314	0.779		
M1	MELD-Na	0.232	0.032	1.261	1.184–1.344
M2	Intercept (α)	-4.073	0.558		
M2	AIMS65	1.039	0.167	2.825	2.038–3.918
M3	Intercept (α)	-7.239	0.953		
M3	MELD-Na	0.192	0.034	1.212	1.134–1.295
M3	AIMS65	0.619	0.197	1.857	1.261–2.735
M4	Intercept (α)	-8.742	1.367		
M4	AIMS65	2.171	0.361	8.769	4.318–17.806
M5	Intercept (α)	-13.193	2.189		
M5	Rockall	1.560	0.275	4.760	2.779–8.152
M6	Intercept (α)	-17.184	3.258		
M6	AIMS65	1.754	0.369	5.776	2.803–11.905
M6	Rockall	1.285	0.348	3.614	1.827–7.150

To compute a predicted risk, multiply each predictor value by its β, add the intercept α to obtain η, then transform using the logistic function above.

Internal Validation and Performance Metrics

Discrimination was quantified using the AUROC. Uncertainty was estimated via nonparametric bootstrap resampling (2,000 replicates) with percentile 95% confidence intervals.

Calibration was assessed using calibration-in-the-large (intercept) and calibration slope estimated by regressing observed outcomes on the model linear predictor, with optimism-correction using bootstrap.

Overall accuracy was summarized using the Brier score. Decision-curve analysis (DCA) was computed as net benefit across clinically relevant threshold probabilities. For the ICU endpoint, DCA is interpreted as decision support for ICU triage under institutional criteria (not organ-support necessity).

Software

All analyses were reproduced from the study database using Python (pandas, statsmodels) with maximum-likelihood logistic regression.
