# Correction: Upper Gastrointestinal Bleeding in Cirrhosis: Differential Performance of Risk Scores for In-Hospital Mortality and Intensive Care Unit Triage Decisions

**DOI:** 10.7759/cureus.c438

**Published:** 2026-05-22

**Authors:** Ilie Marius I Ciorba, Nicoleta Craciun Ciorba, Simona M Bataga

**Affiliations:** 1 Internal Medicine, George Emil Palade University of Medicine, Pharmacy, Science, and Technology of Târgu Mureș, Tîrgu Mureș, ROU; 2 Department of Medicine and Pharmacy, Institution Organizing University Doctoral Studies, University of Medicine, Pharmacy, Science and Technology “George Emil Palade”, Tîrgu Mureș, ROU; 3 Gastroenterology, George Emil Palade University of Medicine, Pharmacy, Science, and Technology of Târgu Mureș, Tîrgu Mureș, ROU

This article has been corrected to change Dr. Ilie Ciorba’s second affiliation to: Department of Medicine and Pharmacy, Institution Organizing University Doctoral Studies, University of Medicine, Pharmacy, Science and Technology “George Emil Palade”, Târgu Mureș, ROU.

